# Supplementary material for: Study on repair materials and technologies for addressing crack-related damage in the Earthen City Wall of Kaifeng
Source: PLoS One. 2025 Oct 27;20(10):e0334834. doi: 10.1371/journal.pone.0334834 (PMC12558513; doi:10.1371/journal.pone.0334834)
Supplement: Attachment — Submitted filename: Response to reviewers.pdf [file pone.0334834.s005.pdf]

Responses to reviewers

Dear editor and reviewers,

We really appreciate the time the reviewers have spent on our manuscript. The constructive and helpful suggestions are valuable for us to improve the quality of the manuscript. We have revised the manuscript carefully according to all the comments. All the changes are marked with *highlight* tool in the revised manuscript.

Part I: To Editor

Thank you very much for giving us helpful suggestions. The manuscript has been revised in accordance with all the comments presented by the reviewers. Great thanks to referees and editors for the time and efforts on this manuscript.

Sincerely yours,

Tingting Yue

Part II: to Referee 1

<u>Comment 1:</u> Language and Readability

The manuscript requires thorough English editing to correct grammatical errors, improve clarity, and reduce repetition. Several “Error! Reference source not found.” placeholders remain in the text and must be fixed. Improving the overall readability will significantly strengthen the manuscript’s presentation.

<u>Response 1:</u> Thank you for your valuable feedback regarding the language and readability of our manuscript. I am pleased to inform you that we have thoroughly revised the manuscript to address these concerns. We have corrected all grammatical errors, enhanced the clarity of the text, and eliminated any repetition. Additionally, we have resolved all the “Error! Reference source not found.” placeholders that were present in the text.

<u>Comment 2:</u> Novelty and Positioning

While the experimental program is systematic, the novelty compared to existing studies is not strongly emphasized. Many of the repair materials (quicklime, sodium methylsilicate, polyurethane, styrene-acrylic emulsion) and techniques (EICP/MICP) have already been studied in the context of soil stabilization and conservation. The authors should clearly highlight:

（a）What specific research gap this study addresses.

（b）How the work differs from and advances beyond prior studies.

（c）Whether the innovation lies in the optimization of material ratios, the multi-indicator evaluation method, or the integration of hydraulic and mechanical performance metrics. A deeper comparison with recent international literature is recommended.

<u>Response 2:</u> We sincerely express our gratitude to the reviewers for their profound concern regarding the innovativeness of our research and for providing constructive insights. In the revised version, we have incorporated additional content within the "Introduction" and "Discussion" sections. This is aimed at explicitly emphasizing the

research gaps, innovative aspects, and international comparisons. The specific enhancements are outlined as follows:

(a) Addressing the research gap

The majority of existing research has primarily centered on the application of single repair materials (such as quicklime, sodium methyl silicate, polyurethane, and styrene-acrylic emulsion) or individual techniques (such as MICP/EICP). However, there has been a notable scarcity of systematic evaluations of composite repair materials. In particular, there is a lack of research on crack grouting that takes into account both hydraulic properties (waterproof performance) and mechanical properties (strength). In response to this research gap, the present study designed and conducted tests on 27 combinations of quicklime-soil-admixtures. This effort effectively fills the void in the research regarding the "comprehensive multi-indicator evaluation of composite materials" for the crack repair of earthen heritage sites.

(b) In contrast to prior studies, the advancements of this research are manifested in:

Most of the existing studies predominantly concentrate on the application of single repair materials (such as quicklime, sodium methyl silicate, polyurethane, and styrene-acrylic emulsion) or individual techniques (such as MICP/EICP). However, there has been a dearth of systematic evaluations of composite repair materials. In particular, there is a lack of research on crack grouting that concurrently takes into account both hydraulic properties (waterproof performance) and mechanical properties (strength).

Specifically addressing this shortfall, this research designed and tested 27 combinations of quicklime-soil-admixtures, thereby filling the research gap in the "comprehensive multi-indicator assessment of composite materials" for the repair of cracks in earthen heritage sites.

(c) Origins of Innovation

It was determined that the combination of 7.5% quicklime and 7% sodium methyl silicate is the optimal one, demonstrating excellent comprehensive properties (with a water absorption rate of only 3.9% ± 0.2%). For the first time, the coefficient method was introduced into the restoration of earthen heritage sites, establishing a reusable

comprehensive scoring method. Moreover, the evaluation concept of "integrating strength and waterproof performance" was proposed and verified, offering a new approach for long-term crack repair.

(d) Comparison with International Literature

In the revised manuscript, a comparison with this year's international research has been added from line 125 to line 137. The detailed content is as follows:

Liu et al. carried out research on superhydrophobic coatings for the surface of earthen heritage sites. They emphasized the potential of such coatings in waterproof and moisture-resistant properties. Nevertheless, these materials typically place more emphasis on the surface coverage effect, with relatively less attention given to crack filling repairs and the durability within the structure [23]. Li et al. improved the capillary water rise and water contact angle of silty soil by applying silicone-based waterproofing agents, achieving an inhibition rate of nearly 98%. This research verified the significance of adding a small quantity of silicates in enhancing the waterproof performance [24]. Cheng et al. conducted a systematic evaluation of polyurethane grouting materials. These materials exhibited excellent penetration ability and adhesiveness in crack repair and grouting filling. In particular, their adaptability on complex interfaces was improved. Although this study was aimed at the repair of concrete structures, the use of polymer waterproof agents and organic materials therein provides a reference for soil restoration [25].

<u>**Comment 3:**</u> **Figures and Tables**

Several figures and tables are of low quality, unclear, or missing proper references. All figures should be redrawn at higher resolution with consistent labeling. Broken cross-references must be corrected. Ensure that captions are detailed enough to allow standalone interpretation.

<u>**Response 3:**</u> We sincerely appreciate your patient and meticulous review as well as the insightful revision suggestions.

We have conducted a thorough reexamination and redrawing of all figures. This effort is aimed at ensuring their presentation in a higher resolution, while also

maintaining consistency in the labeling. Moreover, we will meticulously rectify all broken cross-references and enrich the captions of the figures and tables. This is to guarantee that readers can clearly and independently comprehend the information conveyed by each one.

Comment 4: Statistical Analysis and Rigor

The results are largely presented as single values without error bars, standard deviations, or information on replicates. For a journal such as PLOS ONE, statistical robustness is essential. Please clarify the number of replicates for each test and include appropriate measures of variability. If possible, apply statistical tests (e.g., ANOVA) to support claims of significant differences.

Response 4: We sincerely appreciate the valuable feedback from the reviewers. All experiments presented in this paper were parallel experiments, and each experimental group was replicated three times. In the illustrated results section, we have now included error bars, which represent the standard deviation (SD) of the experimental data. This addition is made to offer a visual representation of the data variability, thus providing readers with a more comprehensive understanding of the experimental outcomes.

Comment 5: Discussion Section

The discussion is mainly descriptive. It should go beyond reporting test outcomes and provide deeper insights into why certain combinations performed better. For example, (a) what mechanisms explain the superior performance of 7.5% quicklime + 7% sodium methylsilicate? (b)How do these results align with or contradict existing studies? (c) The authors should also discuss limitations (e.g., scale effects, laboratory vs. field conditions) and the potential implications for conservation practices.

Response 5: We are deeply grateful for the insightful comments and suggestions provided by the reviewers.

(a) In the discussion section, from line 855 to 872, we have added an explanation for the reasons why the combination of "7.5% quicklime + 7% sodium methylsilicate" performed well. The specific content is as follows:

The mechanism lies in that the urease-mediated mineralization reaction of urea and quicklime involves the decomposition of urea to generate CO_3^{2-}, which subsequently reacts with Ca^{2+} in slaked quicklime to form $CaCO_3$. This process has been a key focus in the research on urease-induced mineralization by numerous scholars, with the aim of enhancing the mechanical properties of soil. In this study, the calcium ions generated from the hydration of quicklime were employed as the calcium source. This approach not only circumvented the additional costs associated with the use of calcium chloride but also mitigated the environmental pollution caused by calcium ion leaching. Secondly, this study exploited the principle that the CO_2 generated during urease-mediated mineralization promotes the reaction of sodium methyl silicate. This effectively enhanced the carbonation reaction of sodium methyl silicate, thereby improving its utilization efficiency and further enhancing the waterproof performance of the repair material. Thirdly, the carbonation reaction between slaked quicklime in the repair material and CO_2 in the air is crucial for the improvement of the earthen city wall. The CO_2 produced from the reaction of urease and urea also stimulates the mineralization reaction of slaked quicklime. This process not only strengthens the mechanical properties of the region surrounding the cracks and the repair material but also increases the compactness of the repair material. Consequently, it significantly enhances the effectiveness and efficiency of crack repair and reduces the likelihood of crack recurrence.

(b) These findings align with several existing studies. For instance, the role of quicklime in enhancing the mechanical properties of earthen materials through its hydration and carbonation processes has been well - documented in previous research. The study by Yue et al. (2022) also highlighted the importance of quicklime in improving the strength and durability of earthen structures. Similarly, the use of sodium methylsilicate as a waterproofing agent to improve the hydrophobicity and erosion

resistance of earthen materials has been explored in other studies. However, this study provides a novel insight into the synergistic effect of combining quicklime with sodium methylsilicate and urease - mediated mineralization to achieve both mechanical and waterproofing enhancements.

(c) In lines 887 to 898 of the discussion section, we discussed the limitations of the study and the possible impact on protective measures. The specific content is as follows:

Moreover, there are several practical challenges associated with the on-site application of repair materials. Firstly, cost represents a notable hurdle. Certain admixtures, such as waterborne polyurethane and styrene-acrylic emulsion, come at a relatively high price. Consequently, large-scale utilization may be constrained by budgetary limitations. Secondly, compatibility is a crucial aspect. The disparities in composition, pore structure, and moisture content among the soils of different heritage sites, along with the varying environmental conditions where these sites are situated, can all exert an influence on the permeability and bonding properties of the repair materials. This, in turn, has an impact on the overall repair outcome. Inadequate handling may even give rise to new stress concentrations between the repair materials and the original soil matrix, potentially leading to secondary damage. Therefore, when it comes to promoting and implementing these repair materials, it is imperative to formulate personalized repair plans that take into account the specific environmental conditions of local heritage sites.

Part II: to Referee 3

<u>Comment 1:</u> Abstract: If a graphical abstract has been added , ensure it is clear and visually appealing. The written abstract should concisely summarize the study's objectives, methods, key findings (e.g., 3.9% water absorption for the 5% sodium methylsilicate + 7.5% quicklime group), and implications.

<u>Response 1:</u> We sincerely appreciate your meticulous review of our manuscript and your invaluable suggestions. In response to the revision comments you put forward regarding the abstract and the graphical abstract, we have made the following amendments. Concerning the graphical abstract, we have redesigned and optimized it, as presented in the figure below.

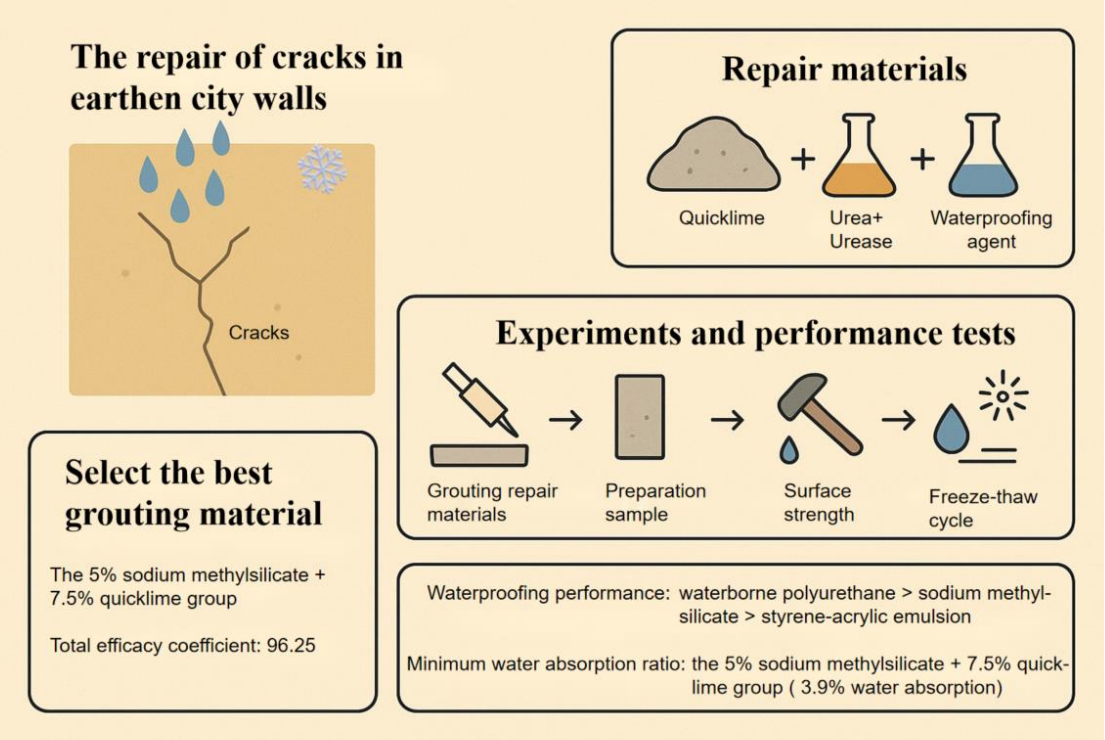

With respect to the textual abstract, we have refined and augmented the abstract part. The detailed content is presented as follows:

Rain and snow seepage into the cracks of the soil wall is the leading cause of its surface weathering, and the key to crack repair lies in developing reasonable repair materials. Based on the carbonation principle and mineralization mechanism of quicklime, this study focuses on the cracks in the city wall of Kaifeng as the research subject. Urea urease solution, quicklime, sodium methylsilicate, styrene-acrylic

emulsion, waterborne polyurethane, and soil were selected to prepare 27 groups of high-fluidity repair materials with varying proportions. The surface strength, water absorption, surface strength after water absorption, consistency and freeze-thaw cycle tests of crack repair samples were carried out to explore the repair effects of different proportions of repair materials on crack diseases. The results demonstrated that the urease urea solution, along with its mineralization reaction with quicklime and sodium methylsilicate, significantly accelerated the chemical interaction between quicklime and sodium methylsilicate, thereby enhancing the mechanical and waterproofing properties of the repair materials. The group composed of 7.5% quicklime and 7% sodium methylsilicate exhibited a total efficacy coefficient of 96.25, indicating superior mechanical strength and waterproofing performance. Among the tested waterproofing agents-sodium methylsilicate, styrene-acrylic emulsion, and waterborne polyurethane-the effectiveness was ranked as follows: waterborne polyurethane > sodium methylsilicate > styrene-acrylic emulsion. Notably, the group containing 5% sodium methylsilicate combined with 7.5% quicklime achieved the lowest water absorption rate of 3.9%. Thirty cycles of crack-filling experiments revealed that the crack resistance of the filling material surpassed that of the original sample, while maintaining excellent integrity with the earthen structure even after freeze-thaw cycles.

Comment 2: Introduction: Strengthen the literature review by citing more recent studies to contextualize the research gap.

Response 2: Thank you very much for the reviewers' comments. In the revised manuscript, a comparison with this year's international research has been added from line 125 to line 137. The detailed content is as follows:

Liu et al. carried out research on superhydrophobic coatings for the surface of earthen heritage sites. They emphasized the potential of such coatings in waterproof and moisture-resistant properties. Nevertheless, these materials typically place more emphasis on the surface coverage effect, with relatively less attention given to crack filling repairs and the durability within the structure [23]. Li et al. improved the capillary water rise and water contact angle of silty soil by applying silicone-based waterproofing

agents, achieving an inhibition rate of nearly 98%. This research verified the significance of adding a small quantity of silicates in enhancing the waterproof performance [24]. Cheng et al. conducted a systematic evaluation of polyurethane grouting materials. These materials exhibited excellent penetration ability and adhesiveness in crack repair and grouting filling. In particular, their adaptability on complex interfaces was improved. Although this study was aimed at the repair of concrete structures, the use of polymer waterproof agents and organic materials therein provides a reference for soil restoration [25].

Comment 3: Methodology : Provide a flowchart or diagram to illustrate the experimental workflow, including sample preparation, testing procedures, and data analysis.

Response 3: We sincerely appreciate your meticulous review of our manuscript and the invaluable suggestions you provided. Regarding your comment that "it is recommended to supplement the methods section with a flowchart or diagram to elucidate the experimental workflow", we have incorporated Figure (Experimental Workflow of crack repair materials) into the revised manuscript.

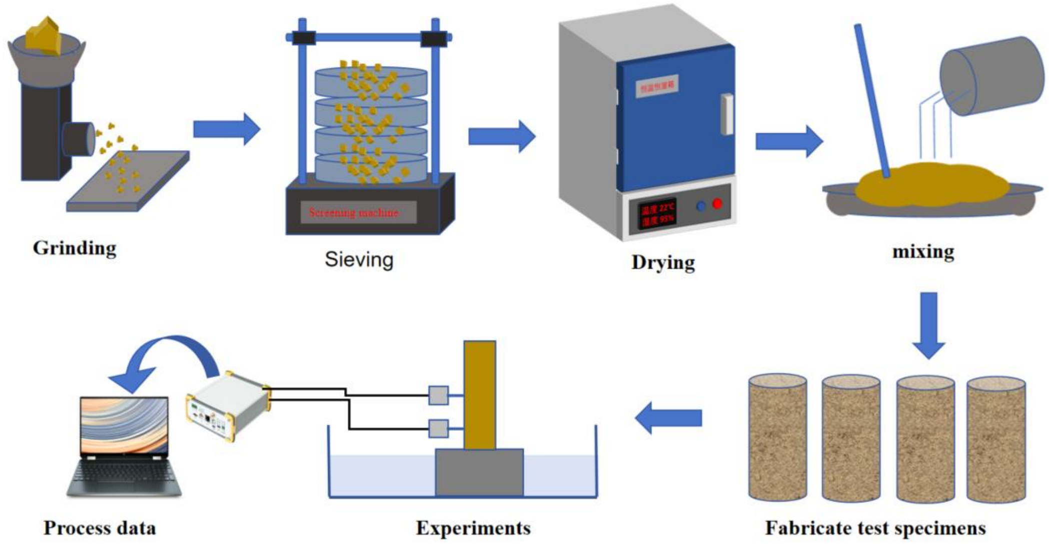

Experimental workflow of crack repair materials.

Flowchart illustrating the experimental methodology, including soil sample

preparation, mixing with quicklime and waterproofing agents, molding and curing, followed by performance testing (consistency, surface strength, water absorption, strength after water absorption, water loss, and freeze-thaw cycles). The results were analyzed using the efficacy coefficient method to determine the optimal mix ratio, which was further validated through simulated crack repair tests under freeze-thaw conditions.

<u>Comment 4:</u> Results : The claim of a 3.9% water absorption rate is significant but needs statistical validation. Discuss why sodium methylsilicate outperforms other waterproofing agents in greater detail.

<u>Response 4:</u> We are sincerely grateful for the insightful comments from the reviewers. In the analysis of the water absorption test within Section 4.2, specifically from line 583 to line 601, we have elaborated on the reasons why sodium methyl silicate outperforms other waterproofing agents. The detailed content is presented as follows:

Sodium methylsilicate reacts with atmospheric carbon dioxide to produce silicic acid (H2SiO3) and carbonate ions (CO32-) or bicarbonate ions (HCO3-). This reaction leads to the formation of an insoluble silicic acid gel on the surfaces of soil particles and within cracks, thereby enhancing the hydraulic properties of soil particles adjacent to fissures in urban earthen walls. Compared to styrene-acrylic emulsion, sodium methylsilicate exhibits a broader penetration range during repair processes and significantly enhances the waterproof performance of soil on both sides of cracks. The hydrophobic layer of the polysilicane film, formed by sodium methylsilicate, requires a combination of CO2 and moisture from the air. Similarly, the carbonization process of quicklime in the atmosphere also necessitates both CO2 and humidity. When the quicklime content is low, the required amounts of CO2 and humidity are reduced. Under these conditions, sodium methylsilicate can react with sufficient CO2 to form a hydrophobic layer of polysiloxane film. The quantity of sodium methylsilicate directly influences the production of films and enhances the waterproofing effect. However, an excessive quicklime content can lead to competition between quicklime and sodium methylsilicate for CO2 and moisture present in the air [36]. This competition ultimately

results in a reduction in the formation of silica films. When quicklime undergoes carbonization to produce calcium carbonate, its volume expands. An excessive amount of quicklime may lead to the formation of micro-cracks in the repair material. Water can infiltrate through these micro-cracks, which is a primary factor contributing to the increased water absorption of the repair material as quicklime content rises.

Comment 5: Discussion : Elaborate on the practical challenges of applying these repair materials in situ, such as cost, scalability, and compatibility with existing structures.

Response 5: We sincerely appreciate the valuable comments from the reviewers. In the "Discussion" section (lines 887-898) of our manuscript, we have incorporated the relevant content, which is presented as follows:

There are several practical challenges associated with the on-site application of repair materials. Firstly, cost represents a notable hurdle. Certain admixtures, such as waterborne polyurethane and styrene-acrylic emulsion, come at a relatively high price. Consequently, large-scale utilization may be constrained by budgetary limitations. Secondly, compatibility is a crucial aspect. The disparities in composition, pore structure, and moisture content among the soils of different heritage sites, along with the varying environmental conditions where these sites are situated, can all exert an influence on the permeability and bonding properties of the repair materials. This, in turn, has an impact on the overall repair outcome. Inadequate handling may even give rise to new stress concentrations between the repair materials and the original soil matrix, potentially leading to secondary damage. Therefore, when it comes to promoting and implementing these repair materials, it is imperative to formulate personalized repair plans that take into account the specific environmental conditions of local heritage sites.

Comment 6: References: Ensure all references (e.g., [1], [25], [40]) are complete

and follow the journal's formatting guidelines.

<u>Response 6:</u> We sincerely appreciate your meticulous review of our manuscript and the invaluable suggestions you provided. Regarding your recommendation that "references must be ensured to be complete and comply with the format requirements of the journal", we have painstakingly checked each reference item by item and adjusted its format. This is to guarantee that every citation is not only complete but also error-free, while strictly adhering to the journal's format specifications. The revised reference list has been updated in the revised manuscript.
