# Supplementary material for: Study on repair materials and technologies for addressing crack-related damage in the Earthen City Wall of Kaifeng
Source: PLoS One. 2025 Oct 27;20(10):e0334834. doi: 10.1371/journal.pone.0334834 (PMC12558513; doi:10.1371/journal.pone.0334834)
Supplement: Attachment — Submitted filename: Response to reviewers.docx [file pone.0334834.s004.docx]

**Responses to reviewers**

Dear editor and reviewers,

We really appreciate the time the reviewers have spent on our manuscript. The constructive and helpful suggestions are valuable for us to improve the quality of the manuscript. We have revised the manuscript carefully according to all the comments. All the changes are marked with *highlight* tool in the revised manuscript.

***Part I: To Editor***

Thank you very much for giving us helpful suggestions. The manuscript has been revised in accordance with all the comments presented by the reviewers. Great thanks to referees and editors for the time and efforts on this manuscript.

Sincerely yours,

Tingting Yue

***Part II: to Referee 1***

**Comment 1: The manuscript presents a valuable study on the repair of cracks in Kaifeng's earth walls using various materials, but there are several areas that require significant improvement before publication. One major concern is the lack of a clear justification for the research’s novelty. While the introduction provides a detailed background on the deterioration mechanisms of earth walls (Page 2, Lines 10–38), it does not sufficiently differentiate this study from previous works. Many references (e.g., Cui et al. [3,4], Zhang et al. [8,9]) discuss similar approaches using strengthening agents, but the authors fail to explicitly state how their methodology or findings contribute to advancing the field. The manuscript would benefit from a stronger argument on what makes this study unique, such as the combination of repair materials or the specific application of the efficacy coefficient method.**

**Response 1:** Thank you for your patient and meticulous review and the suggestions for revisions you provided. We have reorganized and adjusted the introduction, integrating it according to the damage mechanism of the earthen wall, the mineralization restoration technology, and the improvement of the hydrological properties of the earthen site. We made revisions from line 45 to line 80 in the manuscript. The specific revisions are as follows:

The following content has been deleted:~~Cui Kai~~ ^~~[3-4]~~^ ~~and others found that as the dissolution amounts of Na~~_~~2~~_~~SO~~_~~4~~_ ~~and NaCl increase, the mechanical properties of the soil at the site would first decline. As the NaCl content continues to rise, the plasticity and wind erosion resistance of the site soil also decrease. Wind erosion is also one of the main reasons for the damage to the earthen city walls. Wind carrying sand as the driving force repeatedly erodes the surface of the site, causing honeycomb-like patterns to appear on the surface, leading to thinning or even peeling of the surface layer and accelerating the damage. Yan Gengsheng~~ ^~~[5]~~^ ~~believes that the particle size range of the earthen city walls that are easily eroded by wind is mainly between 0.05 and 0.25mm, and when the wind speed reaches 7 to 10 m/s, the damage to the earthen site is the most severe. Through the study of wind erosion volume, Chen Wenwu~~ ^~~[6]~~^ ~~and others found that the erosion volume of the walls on different sides of the windward face is different, and the erosion volume increases significantly within about 30°of the main wind direction. Mao Xiaofei~~ ^~~[7]~~^ ~~analyzed the mechanism and believed that the erosion of the earthen city walls by wind is essentially the blowing and transportation of small particles on the surface layer of the soil under the action of wind force. Sunlight can also cause damage to the earthen city walls. Zhang Huyuan~~ ^~~[8]~~^ ~~conducted indoor simulation and outdoor investigation on the city walls of the Ming Dynasty and found that the thermal conductivity and thermal diffusion coefficients of the surface layer and the parent wall are different, resulting in a temperature difference between the composition of the earthen site and the parent wall. Under the continuous effect of this temperature difference, the contact surface is damaged. The uneven distribution of soluble salts inside the earthen city walls also leads to different thermal conductivity coefficients in different parts of the earthen city walls, thereby causing delamination diseases~~ ^~~[9]~~^~~. It can be seen that rain, snow and other "water" seeping into the earthen city walls, under the action of scouring, evaporation, freezing and thawing, cause the strength of the surface layer of the earthen city walls to decline, and phenomena such as powdering and peeling occur.~~

~~Due to the long geological age, low strength and outdoor natural environment of the earthen city walls, while studying the causes of diseases of the earthen city walls, many scholars have also studied the protection technology of the earthen city walls to prevent the peeling of the surface of the earthen city walls, especially the further development of cracks, which has increasingly attracted the attention of researchers. The repair and protection of earthen city walls usually adopt three methods: physical reinforcement, chemical reinforcement and chemical-physical reinforcement. Some researchers have used wooden anchor rods to reinforce the soil, and some scholars have used anchor rods and long filaments of various materials to reinforce the walls of small earthen city walls, all of which have achieved good reinforcement effects. For partially damaged cracks, most choose grouting and applying chemical solutions for repair. For the repair of crack diseases in partially damaged earthen city walls,~~

Add the following content:

Richards employed a portable wind-rain erosion simulation device to investigate the comparative impacts of wind, sand-laden wind, and wind-rain on the degradation of soil-based heritage. Furthermore, he elucidated the correlation between the early degradation characteristics of test walls induced by these factors and the degradation patterns documented on historical city walls ^[3]^. Yue Jianwei et al. employed an X-ray diffractometer and a scanning electron microscope to analyze the chemical composition and microstructure of soil samples from earthen sites. Using the strength reduction method, they determined the minimum safety factor of trenches under both natural conditions and following rainfall exposure. It was found that rainfall erosion, freeze-thaw cycle and man-made destruction were the main factors leading to the degradation of earthen sites ^[4]^. As the water content increases, the safety factor of earthen ruins decreases, and water exerts a significant influence on the mechanical strength of these structures. Soil exhibits large pores, numerous initial cracks, and a tendency toward easy spalling. Richards et al. employed a newly developed cellular automata model to evaluate the risk of environmental degradation over a 100-year period under a range of potential climate and protection scenarios. It has been found that an increase in wind speed significantly elevates the risk of overall deterioration, while the risk associated with rainfall-driven degradation characteristics is even more pronounced ^[5]^. This paper investigates the impact of floods on earthen architectural heritage and proposes a qualitative methodology for assessing the flood vulnerability of such structures. Through a case study of the Iberian Peninsula, this paper analyzes the visible damage to soil structure buildings associated with superstructure-soil interaction, as well as the damage caused by flooding, to emphasize the fragility and resilient resources of this cultural heritage. Finally, a flood control strategy and protection standard for soil architectural heritage are proposed ^[6]^. Cavicchio discovered that local damage, caused by cracks and material losses, rendered the original wall more vulnerable when exposed. Additionally, the intensity of precipitation events has increased as a result of climate change, thereby providing a foundation for practical research on the shelter coat of Union Fort. The optimal properties of repair materials encompass excellent consistency (plasticity), minimal shrinkage, strong adhesion to the substrate (durability), high cohesive strength against erosion (durability), low liquid water absorption, high desorption capacity, moderate water vapor permeability, and equivalence in color and texture to the substrate ^[7]^. It is evident that precipitation, such as rain and snow, infiltrates into the soil city wall, leading to a reduction in the strength of its surface layer. Under the combined effects of scouring, evaporation, freezing, and thawing, this results in pulverization and spalling of the wall.

Due to its long geological age, low strength, and exposure to an outdoor natural environment, the earth wall is susceptible to surface degradation, particularly the further development of cracks, which has increasingly drawn the attention of researchers.

**Comment 2: （a）The methodology section lacks crucial details that would allow replication. For example, while the authors describe the preparation of soil samples and repair materials (Page 6, Lines 12–40), they do not provide sufficient information about the curing conditions, environmental parameters, or the exact procedure for mixing and applying the materials. （b）Additionally, while the freeze-thaw cycle test is mentioned (Page 12, Lines 4–18), the manuscript does not specify the temperature range, cycle duration, or the reasoning behind the number of cycles chosen. Given that freeze-thaw durability is a key performance indicator for long-term crack repair, these missing details significantly weaken the reliability of the results. The authors should provide explicit temperature settings and cycle descriptions, referencing standard testing protocols where applicable.**

**Response 2:** Thank you for the reviewers' comments. We have made detailed additions and improvements to the “3.1 Experimental Design” and “3.2 Sample Preparation” sections.

(a) **Here is the revised section “3.1 Experimental Design” and “3.2 Sample Preparation” :**

### **3.1 Experimental design**

To ensure the test has broad applicability, the primary component of the repair material is silty clay. The waterproofing agents incorporated include sodium methylsilicate, styrene-acrylic emulsion, and waterborne polyurethane. Additionally, the water utilized consists of urease solution and urea solution. In conjunction with the commonly utilized quicklime content, quicklime is selected at concentrations of 7.5%, 15%, and 22.5% ^[26]^. To facilitate a more effective comparison of the performance differences among various components, the dosages of the waterproofing agent are set at 3%, 5%, and 7%. Additionally, both the urease and urea solutions are maintained at a concentration of 1 mol/L. According to the literature, the moisture content of grouting materials typically ranges from 53% to 58%. Considering the characteristics of silty clay, a moisture content of 55% has been selected as the optimal level for this experiment to ensure adequate fluidity. The testing scheme is presented in Table 4 below:

### **3.2 Sample preparation**

Prior to the experiment, silty clay collected near the earthen city wall will be sun-dried, crushed, sieved through a 2 mm mesh, and dried in an oven. Quicklime will then be added at varying proportions and mixed thoroughly. The mixture will be allowed to rest for 24 hours to ensure complete reaction between quicklime and soil. A solution of urease, urea, and water in a ratio of 1:1:2 will be prepared. Subsequently, different types and concentrations of waterproofing agents will be incorporated into the solution and mixed uniformly. This solution will then be added to varying amounts of quicklime-soil mixtures and stirred for 3 minutes to ensure homogeneity. The resulting mixtures will be poured into standard molds, vibrated for 30 seconds to eliminate air bubbles and ensure uniform material distribution, and subsequently cast into specimens. Each group will consist of three parallel specimens. After preparation, the specimens will be cured for 7 days under controlled conditions. Following curing, surface strength tests, water absorption tests, post-water absorption surface strength tests, and water loss tests will be conducted on the specimen surfaces. Once the data has been analyzed, the optimal repair material ratio will be determined using the effectiveness coefficient method, and crack repair trials will be performed. Cracks will first undergo pre-treatment (e.g., moistening the surrounding area), followed by the injection of repair materials to ensure complete filling of the cracks. Upon completion of the repair, the specimens will be transferred to a curing chamber for an additional 7-day curing period. The curing chamber will maintain a temperature of (20±2)°C and a relative humidity exceeding 95%. During this period, specimens will be misted daily to ensure continuous moisture retention without water accumulation.

**（b）Here is the revised section “3.3 (5) Freeze-Thaw Cycle Test”:**

### **(5) Freeze-thaw cycle test**

After repairing the simulated crack samples, we aim to assess the durability of the repaired materials in a real-world environment. Based on the temperature fluctuations observed in Kaifeng, we have established a freeze-thaw cycle temperature range from -10℃ to 20℃. Each cycle is designed to last for 24 hours, comprising 12 hours of freezing followed by 12 hours of thawing. A total of 30 freeze-thaw cycles were conducted to simulate the extreme winter weather conditions in the Kaifeng area. Surface changes were recorded after each cycle to identify the most suitable material for repairing cracks in earthen structures.

**Comment 3: The discussion of results lacks depth in terms of explaining the chemical and physical interactions between repair materials and the soil. For instance, while the manuscript explains that sodium methylsilicate forms a hydrophobic film (Page 10, Lines 30–38), it does not explore whether this film affects the soil’s permeability or long-term stability. Additionally, the interaction between quicklime, urease, and urea in forming calcium carbonate (Page 7, Lines 20–38) is described, but the implications of this reaction on the material’s porosity and potential shrinkage are not addressed. Furthermore, no information is provided on possible secondary effects, such as efflorescence, which could impact the aesthetic and mechanical properties of the repaired cracks. A more detailed chemical discussion is needed to strengthen the conclusions.**

**Response 3:** Thank you for your patient and meticulous review as well as the suggestions for revisions you provided.

The objective of this study is to develop a material for repairing cracks in earthen ruins. The desired repair material should exhibit excellent fluidity, effective water-blocking properties, resistance to cracking, sufficient mechanical strength, and a color that closely matches the original soil wall. To achieve these characteristics, it is necessary to combine multiple materials into a composite system. Based on preliminary experiments and literature reviews, while considering the requirements for cultural heritage preservation, we selected two traditional materials commonly used in the construction of soil walls: soil and quicklime. Additionally, sodium methylsilicate was incorporated due to its superior water-blocking capabilities. Sodium methylsilicate reacts with atmospheric carbon dioxide to produce silicic acid (H₂SiO₃) and carbonate ions (CO₃²⁻) or bicarbonate ions (HCO₃⁻), forming insoluble silicic acid gel on the surfaces of soil particles and within cracks. This process enhances the hydrological stability of the soil particles surrounding the cracks. Compared to phenolic-acrylic emulsions, sodium methylsilicate exhibits a broader penetration range during application, thereby more effectively improving the waterproofing performance of the soil adjacent to the cracks.

After quicklime is mixed with water and undergoes chemical reaction, the Ca²⁺ions in the generated calcium hydroxide will react with the CO_3_^2-^ ions formed by the carbonization of sodium methyl silicate and the CO_3_^2-^ ions formed by the mineralization of urease, thereby generating CaCO₃and enhancing the mechanical properties of the repair material.

The mineralization reaction of urease with urea and slaked quicklime involves the decomposition of urea to produce CO_3_^2-^, which subsequently reacts with Ca^2+^ from slaked quicklime to form CaCO_3_. This process represents a critical focus in many scholars' research on urease mineralization, with the primary objective being the enhancement of soil mechanical properties. In this study, calcium ions derived from the hydration of quicklime, a principal material in soil sites, are utilized as the calcium source. This approach not only avoids the additional costs associated with calcium chloride but also mitigates environmental pollution caused by calcium ion loss. Furthermore, the study leverages the principle that CO_2_ generated during urease mineralization promotes the carbonation reaction of sodium methyl silicate, thereby enhancing its carbonation efficiency and improving its utilization. Additionally, varying amounts of slaked quicklime exist within the soil wall, where the carbonation reaction between slaked quicklime and atmospheric CO_2_ serves as the fundamental mechanism for strengthening the soil wall. The CO_2_ produced by the interaction of urease and urea further facilitates the mineralization reaction of slaked quicklime, thereby improving the mechanical properties of both the crack surroundings and the repair materials, increasing the density of the repair materials, enhancing the effectiveness and efficiency of crack repair, and reducing the likelihood of crack formation.

The effectiveness of the crack repair was validated through practical sealing experiments. Given its large scale and construction based on traditional techniques, the earthen city wall inherently possesses a relatively high level of safety. However, the primary cause of damage to the earthen city wall is the increase in moisture content within the cracked sections, primarily induced by factors such as rainfall. Preventing water infiltration along the cracks into the interior is a critical task for preserving the structural integrity of the city wall. Consequently, the integrity and water-blocking capability after crack repair serve as key indicators for assessing the quality of the repair. Based on the results of the sealing experiments, the crack repair material developed by the research group demonstrates excellent filling properties, minimal shrinkage, and a color that closely matches that of the earthen city wall.

The carbonization reaction formula of methylsilicate acid sodium：

*CH*3*SiO*3*Na*+*CO*2+*H*2*O*→*CH*3*Si*(*OH*)3+*NaHCO*3
The mineralization reaction formula of urease：

$$CO(NH_{2})_{2}+H_{2}O\to2NH_{3}+CO_{2}$$

$$2NH_{3}+2H_{2}O\to2NH_{4}^{+}+2OH^{-}$$

$$CO_{2}+H_{2}O\to H{CO}_{3}^{-}+H^{+}$$

$$H{CO}_{3}^{-}+H^{+}+2OH^{-}\to{CO}_{3}^{2-}+2H_{2}O$$

$$Ca^{2+}+{CO}_{3}^{2-}\to CaCO_{3}\downarrow$$

The specific modifications are as follows:

(a) **We add the following content from line 533 to line 537 of the manuscript:**

After the quicklime is hydrated with water, the calcium ions (Ca^2+^) from calcium hydroxide will react with carbonate ions (CO_3_^2-^) generated through the carbonization of sodium methylsilicate and those produced by the mineralization of urease. This reaction leads to the formation of calcium carbonate (CaCO_3_), thereby enhancing the mechanical properties of remediation materials.

(b) **We will insert the following content starting from line 579 to line 584 of the manuscript:**

Sodium methylsilicate reacts with atmospheric carbon dioxide to produce silicic acid (H_2_SiO_3_) and carbonate ions (CO_3_^2-^) or bicarbonate ions (HCO_3_^-^). This reaction leads to the formation of an insoluble silicic acid gel on the surfaces of soil particles and within cracks, thereby enhancing the hydraulic properties of soil particles adjacent to fissures in urban earthen walls. Compared to styrene-acrylic emulsion, sodium methylsilicate exhibits a broader penetration range during repair processes and significantly enhances the waterproof performance of soil on both sides of cracks.

(c) **We will add the following content from line 826 to line 844 of the manuscript:**

The properties of earthen site restoration materials are primarily influenced by the chemical reaction products and reaction kinetics of urease, urea, quicklime, and sodium methylsilicate. The mineralization reaction of urease with urea and quicklime involves the decomposition of urea to produce CO_3_^2-^ and CaCO_3_ in conjunction with Ca^2+^ from hydrated quicklime. This process constitutes a key area of investigation for many scholars studying urease mineralization as a means to enhance the mechanical properties of soil. In this study, calcium ions generated during the maturation of quicklime are employed as a calcium source. This approach not only eliminates the additional cost associated with the use of calcium chloride but also prevents environmental pollution caused by calcium ion leaching. Furthermore, this study leverages the principle that CO_2_ produced during urease mineralization facilitates the reaction of sodium methylsilicate, thereby enhancing the carbonation reaction of sodium methylsilicate, improving its utilization efficiency, and strengthening the waterproofing performance of the repair materials. Thirdly, the presence of hydrated quicklime in the repair materials plays a critical role. The carbonation reaction between hydrated quicklime and atmospheric CO_2_ serves as the foundation for enhancing the soil wall's properties. Additionally, the CO_2_ generated from the urease-urea reaction further promotes the mineralization reaction of hydrated quicklime, thereby improving the mechanical properties near cracks and within the repair materials, increasing the compactness of the repair materials, enhancing the effectiveness and efficiency of crack repair, and reducing the likelihood of crack formation.

**Comment 4: There are several inconsistencies and grammatical errors throughout the manuscript. For instance, "methicosilicate" appears multiple times (Page 3, Line 20; Page 8, Line 5) but is likely meant to be "methylsilicate." Similarly, "waterborne polyurethane" and "water-based polyurethane" are used interchangeably (Page 3, Line 15; Page 9, Line 30), which could confuse readers. Terminology should be consistent throughout the paper. The abstract (Page 1, Lines 10–25) also contains awkward phrasing, such as "the repair material does a great job fixing the simulated crack," which should be revised for a more formal academic tone. Furthermore, certain sentences are overly informal, such as "this stuff is pretty strong" (Page 2, Line 14), which should be rewritten in a more precise scientific manner.
Response 4:** We are extremely grateful for the reviewers' comments. Methicosilicate is indeed a miswriting of methylsilicate. We have rechecked and revised the grammar and structural details in the paper.

**Comment 5: Figures and tables require improvements in clarity. Some figures, such as the particle gradation diagram (Page 7, Figure 1), lack clear axis labels, making it difficult to interpret trends. Tables summarizing experimental data should include statistical comparisons to highlight significant differences between groups. Additionally, some references in the bibliography appear incomplete or improperly formatted. For example, certain citations (e.g., Page 19, References [12,13]) lack full journal details and DOI links. Ensuring correct formatting and consistency across citations is essential for maintaining academic credibility.**

**Response 5:** We are extremely grateful for the reviewers' comments. We have rechecked and revised the charts and numerical details in the paper. Due to the lack of DOI information for some references, there are no DOI links for those references.

***Part Ⅲ: to Referee 2***

**Comment 1: A graphical abstract should be included in the manuscript to make it more appealing.**

**Response 1:** We sincerely appreciate the valuable feedback provided by the reviewers. Presented below is the graphical abstract of this study.

**Comment 2: The abstract is too vague and does not summarize key findings properly.**

**Response 2:** We are extremely grateful for the reviewers' comments. We have re-examined and revised the summary content of the paper.

**Abstract:** Rain and snow seepage into the cracks of soil wall is the main cause of its surface weathering, and the key to crack repair lies in developing reasonable repair materials. Based on the carbonation principle and mineralization mechanism of quicklime, this study focuses on the cracks in the city wall of Kaifeng as the research subject. Urea urease solution, quicklime, sodium methylsilicate, styrene-acrylic emulsion, waterborne polyurethane, and soil were selected to prepare 27 groups of high-fluidity repair materials with varying proportions. The surface strength, water absorption, surface strength after water absorption, consistency and freeze-thaw cycle tests of crack repair samples were carried out to explore the repair effects of different proportions of repair materials on crack diseases. The results demonstrated that the urease urea solution, along with its mineralization reaction with quicklime and sodium methylsilicate, significantly accelerated the chemical interaction between quicklime and sodium methylsilicate, thereby enhancing the mechanical and waterproofing properties of the repair materials. The group composed of 7.5% quicklime and 7% sodium methylsilicate exhibited a total efficacy coefficient of 96.25, indicating superior mechanical strength and waterproofing performance. Among the tested waterproofing agents—sodium methylsilicate, styrene-acrylic emulsion, and waterborne polyurethane—the effectiveness was ranked as follows: waterborne polyurethane > sodium methylsilicate > styrene-acrylic emulsion. Notably, the group containing 5% sodium methylsilicate combined with 7.5% quicklime achieved the lowest water absorption rate of 3.9%. Thirty cycles of crack-filling experiments revealed that the crack resistance of the filling material surpassed that of the original sample, while maintaining excellent integrity with the earthen structure even after freeze-thaw cycles.

**Comment 3: The manuscript addresses an important topic, but its novelty needs to be clearly stated.**

**Response 3:** We are extremely grateful for the comments from the reviewers. This paper conducts a systematic study on the repair materials and techniques for the cracks in the ancient city walls of Kaifeng. Its novelty mainly lies in the following aspects:

(a) **Innovation in the formulation of repair materials**

This paper uses silty clay as the main material and combines various materials such as quicklime, methylsilicate, styrene-acrylic emulsion, and waterborne polyurethane to construct a multi-material composite system. This composite system integrates the advantages of inorganic materials (such as quicklime and methylsilicate) and organic materials (such as styrene-acrylic emulsion and waterborne polyurethane). It not only utilizes the durability and stability of inorganic materials but also benefits from the adhesion and waterproofing properties of organic materials, significantly enhancing the comprehensive performance of the repair material. The introduction of urea-urease solution in the repair material enables the catalytic decomposition of urea by urease to generate calcium carbonate precipitation, thereby improving the strength and durability of the repair material. This bio-induced calcium carbonate precipitation technology is rarely applied in the restoration of earthen walls, providing a new idea for the improvement of the performance of the repair material.

(b) **Innovation in testing methods and evaluation systems**

This study employs the efficacy coefficient method to comprehensively evaluate the performance of 27 groups of repair materials. This approach integrates multiple indicators, including surface strength, water absorption, consistency, strength after water absorption, and water loss rate. By assigning different weights to these indicators, it provides a scientific assessment of the overall performance of each material group. This method not only overcomes the limitations of single-indicator evaluation but also allows for flexible weight adjustments based on regional environmental conditions and specific requirements, thereby facilitating the selection of the most appropriate repair materials. To validate the practical application of the repair materials, this study simulates the crack environment of the Kaifeng city wall by artificially creating cracks and subsequently repairing them. The durability and compatibility of the repair materials with the soil are then assessed through freeze-thaw cycle tests. This testing method, which closely mimics real-world working conditions, offers a more accurate reflection of the repair materials' performance in actual use, providing a robust scientific basis for their practical application.

(c) **Innovation in repair concepts and technologies**

Traditional materials for repairing earthen walls have often prioritized strength enhancement while overlooking durability. In the development of repair materials, this study not only enhances the mechanical properties through the incorporation of quicklime and organic materials but also significantly improves durability by leveraging the waterproof characteristics of substances such as methylsilicate and water-based polyurethane. This approach, which balances mechanical performance with durability, offers a more holistic solution for addressing cracks in earthen walls. During the actual grouting process, this study introduces an optimized pre-treatment procedure for cracks (e.g., moistening the areas surrounding the cracks), effectively resolving the issue of rapid water loss in dry soil that can lead to material cracking. This improvement enhances both the adhesion between the repair materials and the soil, as well as the overall repair efficacy.

**Comment 4: There is a lack of clear objectives; these should be explicitly stated.**

**Response 4:** We are deeply appreciative of the insightful comments provided by the reviewers. The primary objective of this study is to address the cracks and damages in the Kaifeng earthen city wall using highly fluid repair materials for restoration. Unlike previous studies, this research systematically investigates the synergistic effects of various additives (e.g., urea-urease solution, quicklime, methylsilicate, styrene-acrylic emulsion, water-based polyurethane, etc.). Through experimental analysis, it evaluates the impact of different material mix ratios on crack repair efficacy and identifies the optimal mix ratio. Furthermore, this study employs the efficacy coefficient method to comprehensively assess the performance indicators of the repair materials. This approach enables the adjustment of indicator weights based on specific requirements, thereby providing a more scientific evaluation of the overall performance of the repair materials. By simulating the actual environmental conditions of the Kaifeng earthen city wall, this study experimentally validates the repair materials' durability, compatibility, and performance under freeze-thaw cycles.

**Comment 5: There are a lot of headings in the manuscript, which does not make sense. These should be removed, and flow and coherence should be maintained.**

**Response 5:** We are extremely grateful for the reviewers' comments. We have re-examined and revised the title of the paper.

**Comment 6: The results are not well-organized and should be presented more systematically.**

**Response 6:** We are extremely grateful for the reviewers' comments. We have re-examined and revised the experimental results section of the paper carefully, and the specific modifications have been marked in the manuscript.

**Comment 7: The authors should provide a stronger closing statement.**

**Response 7:** We are extremely grateful for the reviewers' comments. We have carefully reviewed and revised the conclusion section of the paper. The specific modifications are as follows:

**To develop repair materials for soil wall cracks with good fluidity, water resistance, no cracking, high strength, and a color similar to the soil wall, this study investigates the surface strength, water absorption, post-water absorption surface strength, and consistency of 27 groups of high-flow repair materials. These materials vary in quicklime content, sodium methylsilicate concentration, styrene-acrylic emulsion, and waterborne polyurethane solution. Additionally, the practical repair effectiveness of these high-flow materials was verified. The following conclusions were drawn:**

**(1) The mineralization of urease urea in combination with quicklime and sodium methylsilicate accelerates the chemical reaction between quicklime and sodium methylsilicate, thereby enhancing the mechanical and waterproofing properties of restoration materials. This process offers a novel approach for developing materials suitable for soil wall restoration.**

**(2) Sodium methylsilicate, styrene-acrylic emulsion, and waterborne polyurethane can enhance the consistency of fluidized repair materials, whereas quicklime serves to decrease material consistency. With a specific quicklime content, the consistency of repair materials increases as the concentration of sodium methylsilicate and waterborne polyurethane rises, yet decreases with an increase in the concentration of styrene-acrylic emulsion.**

**(3) All three solutions enhance the waterproof performance of high-flow repair materials. In terms of overall waterproof performance, waterborne polyurethane outperforms sodium methylsilicate, which in turn surpasses styrene-acrylic emulsion. The lowest water absorption rate, at 3.9%, was observed in the group with a 5% sodium methylsilicate concentration and a 7.5% quicklime content. As the quicklime content increases, the waterproof performance of each group declines to varying degrees; however, all groups still exhibit relatively high waterproof performance.**

**(4) Quicklime, styrene-acrylic emulsion, and waterborne polyurethane solution significantly enhance the strength of high-fluidity repair materials. Under the same concentration, the surface strength initially increases and subsequently decreases with the increasing quicklime content. When the quicklime content is held constant, the surface strength increases with the increasing concentration of styrene-acrylic emulsion and initially increases before subsequently decreasing with the increasing concentration of waterborne polyurethane.**

**(5) The overall properties of the 27 groups of high-flow restoration materials vary. When in use, considering the differing environmental, climatic, and groundwater conditions across regions, the efficiency coefficient method can be employed to adjust the weighting of various properties, thereby identifying the optimal restoration materials and enhancing the crack restoration of immovable cultural relics in diverse regions. The total efficacy coefficient of the group containing 7.5% quicklime and 7% sodium methylsilicate is 96.25, indicating that it possesses excellent mechanical properties and waterproof performance.**

**In the joint filling test, water should be added to moisten the cracks, ensuring they are not in a dry state, thereby enhancing the bonding strength and restoration efficacy between the repair materials and the earthen site. Thirty crack filling experiments demonstrate that the crack resistance of the filling materials is superior to that of the samples, and the filling materials exhibit enhanced resistance to freeze-thaw cycles. Currently, the research primarily focuses on the development of repair materials, with experimental studies conducted on indoor samples. In the future, it will be necessary to investigate the influence of various factors (such as temperature, humidity, chemical erosion, etc.) on the performance of crack repair materials based on the environmental conditions of earthen sites, thereby enabling a more comprehensive evaluation of material performance in real-world scenarios. A long-term durability test should also be conducted to assess the performance changes of crack repair materials during extended use, including the effects of aging, erosion, and other relevant factors.**

**Comment 8: The aesthetics could be improved in all of the images and graphs.**

**Response 8:** We are extremely grateful for the reviewers' comments. We have re-examined and revised all the figures in the paper carefully.

**Comment 9: All the headings should be revised and made more TO THE POINT and brief.**

**Response 9:** We are extremely grateful for the reviewers' comments. We have re-examined and revised all the titles in the paper.

**Comment 10: All the figures need revision. All the figures have the potential that a lot of information may be included in them. They should be made more informatically appealing with more attention to detail.**

**Response 10:** We are extremely grateful for the reviewers' comments. We have once again carefully rechecked and revised the numerical details in the paper.

**Comment 11: The design of experiments (DOE) should be more clearly explained.**

**Response 11:** We are extremely grateful for the reviewers' comments. We have re-examined and revised the design of experiments section. The specific modifications have been marked in the manuscript.

**Comment 12: The conclusion section should be more robust and clearly state the findings of the results obtained. Future scope and work should be included.**

**Response 12:** Thank you for the reviewers' comments. We have added the future research work and research scope at the end of the conclusion. The specific modifications are as follows:

**Currently, the research primarily focuses on the development of repair materials, with experimental studies conducted on indoor samples. In the future, it will be necessary to investigate the influence of various factors (such as temperature, humidity, chemical erosion, etc.) on the performance of crack repair materials based on the environmental conditions of earthen sites, thereby enabling a more comprehensive evaluation of material performance in real-world scenarios. A long-term durability test should also be conducted to assess the performance changes of crack repair materials during extended use, including the effects of aging, erosion, and other relevant factors.**

**Comment 13: The Results and Discussion section should be added and populated as necessary.**

**Response 13:** We are extremely grateful for the reviewers' comments. We have added a “Discussion” section, and the details are as follows:

**As the content of quicklime increases, the consistency of the restoration materials exhibits a downward trend. This is attributed to the water absorption and hydration reactions of quicklime, which result in water evaporation and consequently reduce the fluidity of the materials. Among the groups, the consistency of the sodium methylsilicate group is the highest, whereas that of the waterborne polyurethane group is the lowest. This indicates that sodium methylsilicate plays a significant role in enhancing the fluidity of materials, whereas waterborne polyurethane decreases material fluidity owing to its high viscosity. The consistency of the low quicklime content group is influenced by the waterproofing agent content, whereas the consistency of the high quicklime content group is primarily determined by the quicklime content. This demonstrates that the quicklime content is a dominant factor influencing the fluidity of materials.**

**The incorporation of styrene-acrylic emulsion and waterborne polyurethane markedly enhanced the surface strength of the repair materials, whereas the effect of sodium methylsilicate was relatively limited. In particular, the styrene-acrylic emulsion demonstrated the highest surface strength at a quicklime content of 15%. This may be attributed to the interweaving of the polymer film formed by the styrene-acrylic emulsion and the compounds generated by the quicklime reaction, thereby forming a dense network structure and enhancing the integrity of the material. Waterborne polyurethane demonstrated high surface strength at a quicklime content of 7.5%. However, as the quicklime content increased, the surface strength of waterborne polyurethane initially rose and subsequently declined. This may be attributed to the hydration and carbonation reactions of quicklime, which are induced by volume expansion and lead to the formation of micro-cracks, thereby reducing the surface strength of the material.**

**Sodium methylsilicate, styrene-acrylic emulsion, and waterborne polyurethane can markedly enhance the waterproofing performance of repair materials, with waterborne polyurethane demonstrating the most significant effect. This may be attributed to the fact that waterborne polyurethane forms strong hydrophobic films by linking linear polyurethane macromolecules through chemical bonds, thereby effectively inhibiting water penetration. Upon water absorption, the surface strength of the repairing materials generally decreased; however, the surface strength of the styrene-acrylic emulsion and waterborne polyurethane groups exhibited only a slight decrease. This indicates that these materials are capable of maintaining high strength and exhibiting good durability even after water absorption.**

**The water loss rate of remediation materials is influenced by both the quicklime content and the type of admixture. Specifically, groups with lower quicklime content exhibit a faster water loss rate, whereas those with higher quicklime content demonstrate a slower water loss rate. This may be attributed to the increase in quicklime content, which results in a reduction of soil porosity and increased difficulty in water evaporation. Sodium methylsilicate demonstrates excellent water retention at a dosage of 7%, potentially owing to the limited formation of silicon oxide film during its reaction, thereby providing reduced resistance to water evaporation.**

**In the actual repair process, it is essential to pretreat cracks (e.g., by wetting the areas surrounding the cracks). This step can minimize the water absorption of grouting materials on both sides of the cracks, allow the moisture in the materials to evaporate normally, and prevent the occurrence of dry cracking. Following pretreatment, the repair material maintains good integrity within the crack, indicating that pretreatment is a critical step in enhancing the repair effectiveness.**

**After 30 freeze-thaw cycles, the water absorption of the repair material at the crack was significantly lower than that of the surrounding soil, and no new cracks appeared in the repair material. This indicates that the repair material exhibits excellent durability and compatibility with the soil. Furthermore, the repair material maintained robust integrity after the freeze-thaw cycles, demonstrating its high reliability and durability in practical applications.**

**Comment 14: The limitations and the future potentials of the study are not sufficiently addressed or acknowledged.**

**Response 14:** Thank you for the reviewers' comments. Here is a detailed supplementary explanation regarding this issue:

This study has yielded significant insights into the development of repair materials and techniques for addressing cracks in the Kaifeng earth city walls. Nevertheless, several limitations warrant acknowledgment. First, the majority of the research was conducted under controlled indoor laboratory conditions. While the performance of the repair materials was assessed by simulating climatic conditions specific to the Kaifeng region (e.g., freeze-thaw cycles), the actual natural environment of the earth city walls is far more complex, encompassing multifaceted interactions such as weathering, biological erosion, and chemical degradation. Consequently, the applicability of the indoor experimental results to real-world scenarios may be constrained. Second, the current investigation primarily emphasizes short-term mechanical properties and waterproofing performance, including surface strength and water absorption. However, a comprehensive evaluation of long-term durability issues, such as material aging and environmental erosion, remains lacking. Additionally, the repair of cracks in earth city walls is influenced not only by individual factors but also by the coupled effects of multiple variables, such as temperature fluctuations, humidity levels, and groundwater dynamics. Although this study incorporated some of these factors, further exploration is needed to fully understand their collective impact and interaction mechanisms.

Despite the aforementioned limitations, this study demonstrates significant potential for future research and practical applications. Moving forward, we plan to investigate the performance of repair materials under multi-factor coupling conditions, accounting for the synergistic effects of temperature, humidity, chemical erosion, and other environmental factors. By conducting experiments under simulated conditions that closely replicate real-world scenarios, we aim to comprehensively evaluate the long-term durability and adaptability of repair materials in complex environments. Additionally, we will carry out long-term durability tests to systematically assess how repair materials degrade or evolve under varying environmental conditions, thereby providing a robust scientific foundation for their practical implementation. Furthermore, we intend to explore the ecological implications of repair materials by examining their interactions with soil microorganisms, vegetation growth, and other ecological components. Through ecological toxicology experiments and field monitoring, we will ensure that the selected materials enhance the structural stability of earth walls without adversely affecting the surrounding ecosystem. We will also investigate innovative repair materials, such as bio-based composites and nanomaterial-enhanced formulations, to further optimize their mechanical properties and environmental compatibility. Finally, leveraging advanced digital technologies (e.g., 3D modeling, drone-based inspections) and intelligent systems (e.g., self-healing materials, smart monitoring platforms), we will develop more precise and adaptive methodologies for crack repair in earth walls. These efforts will enable real-time monitoring, dynamic assessment, and proactive maintenance, ensuring the timely restoration and sustained preservation of earth wall structures.

We are fully cognizant of the limitations inherent in our research and consider these as valuable opportunities for future investigations. In our follow-up work, we will undertake a more rigorous examination of these limitations and provide a more thorough discussion within the paper to enhance both the scientific rigor and practical applicability of our findings. Once again, we extend our sincere gratitude for your insightful feedback and eagerly anticipate your continued guidance.
