# Supplementary material for: Study on repair materials and technologies for addressing crack-related damage in the Earthen City Wall of Kaifeng
Source: PLoS One. 2025 Oct 27;20(10):e0334834. doi: 10.1371/journal.pone.0334834 (PMC12558513; doi:10.1371/journal.pone.0334834)
Supplement: Attachment — Submitted filename: review.docx [file pone.0334834.s003.docx]

**Manuscript Review: Study on Repair Materials and Technologies for Addressing Crack-Related Damage in the Earthen City Wall of Kaifeng**

**Overall Assessment**:
This manuscript presents a valuable investigation into the development and evaluation of repair materials for addressing crack-related damage in the earthen city wall of Kaifeng, a significant cultural heritage site. The study systematically explores the use of traditional materials (soil, quicklime) and waterproofing agents (urea-urease, sodium methylsilicate, styrene-acrylic emulsion, and waterborne polyurethane) to enhance the mechanical and hydraulic properties of repair materials. The experimental approach, including tests for water absorption, surface strength, and freeze-thaw durability, is commendable. However, the manuscript has several areas that require significant revision to improve clarity, scientific rigor, and overall presentation. I recommend **major revisions** before considering it for publication.

**Strengths**

1. **Relevance and Novelty**:
   The study addresses a critical issue in the preservation of earthen cultural heritage sites, which are vulnerable to environmental degradation such as rainfall erosion and freeze-thaw cycles. The focus on high-fluidity repair materials and the synergistic effects of additives like urea-urease and sodium methylsilicate is innovative and relevant to the field of heritage conservation.
2. **Comprehensive Experimental Design**:
   The manuscript describes a thorough experimental setup, testing 27 groups of repair materials with varying compositions (quicklime content, sodium methylsilicate, styrene-acrylic emulsion, and waterborne polyurethane). The inclusion of multiple performance indicators (e.g., water absorption, surface strength, consistency, and freeze-thaw durability) provides a robust evaluation framework.
3. **Practical Implications**:
   The study identifies a promising formulation (5% sodium methylsilicate combined with 7.5% quicklime) that achieves a low water absorption rate (3.9%) and demonstrates superior crack resistance after 30 cycles of testing. These findings have practical implications for the restoration of earthen structures, particularly in regions like Kaifeng with significant environmental challenges.
4. **Use of Analytical Techniques**:
   The application of XRF spectrum analysis to determine the elemental composition of materials is a strength, as it supports the non-destructive characterization of repair materials.

**Weaknesses**

1. **Clarity and Structure**:
   - The manuscript suffers from poor organization and lack of clarity in several sections. The introduction and literature review are not well-articulated, making it difficult to understand the research gap and the specific contribution of this study.
   - Key sections, such as the methodology and results, are fragmented and contain repetitive or incomplete information (e.g., repeated references to "267" or "5.5.5.5" in the OCR output, which may indicate formatting or transcription errors).
   - The abstract and graphical abstract (if included) are not mentioned in the provided excerpts, making it challenging to assess the study's overview.
2. **Formatting and Presentation Issues**:
   - The manuscript contains numerous formatting errors, such as inconsistent mathematical expressions (e.g., "$\mathrm{0} \mathrm{0} \mathrm{0}$" or "$\mathrm{J} \mathrm{J} \mathrm{J}$"), incomplete tables, and missing figure captions or axis labels . These issues hinder the reader's ability to interpret the data.
   - References are incomplete or improperly formatted (e.g., missing DOI links for some citations). This reduces the credibility and traceability of the cited works.
3. **Methodological Details**:
   - The design of experiments (DOE) is not clearly explained. For instance, the rationale for selecting specific concentrations (7.5%, 15%, 22.5%) of quicklime and waterproofing agents is not justified.
   - The sample preparation process is described but lacks details on the standardization of procedures, such as the exact conditions for sun-drying or crushing the clay.
   - The efficacy coefficient method used for evaluating the 27 groups of repair materials is mentioned but not sufficiently detailed, making it difficult to assess its validity or applicability.
4. **Data Presentation and Analysis**:
   - The results section lacks statistical comparisons to highlight significant differences between groups. For example, the water absorption rate of 3.9% for the 5% sodium methyl silicate and 7.5% quicklime group is impressive, but no statistical tests are provided to confirm its significance compared to other formulations.
   - Tables (e.g., Table 5) and figures are referenced but not fully presented in the excerpts, making it challenging to evaluate the data's completeness or clarity.
   - The discussion of results is superficial in places, with limited explanation of why certain formulations (e.g., waterborne polyurethane) outperform others in specific tests.
5. **Response to Environmental Factors**:
   - While the study addresses rainfall erosion and freeze-thaw cycles, it does not sufficiently explore other environmental factors, such as temperature fluctuations, humidity, or groundwater dynamics, which are mentioned as needing further exploration. This limits the study's applicability to real-world conditions.
6. **Language and Grammar**:
   - The manuscript contains grammatical errors and awkward phrasing, likely exacerbated by OCR issues (e.g., "styrene-acylle" instead of "styrene-acrylic"). These errors detract from the manuscript's professionalism and readability.
   - Technical terms and chemical reactions are not consistently formatted, and some equations appear incomplete or incorrectly rendered.

**Recommendations for Improvement**

1. **Improve Clarity and Structure**:
   - Revise the introduction to clearly state the research problem, objectives, and the specific gap this study addresses compared to existing literature.
   - Organize the manuscript into well-defined sections: Introduction, Literature Review, Methodology, Results, Discussion, and Conclusion. Ensure each section flows logically and avoids redundancy.
   - Include a graphical abstract, as suggested by Reviewer 2, to provide a visual summary of the study's key findings and methodology.
2. **Enhance Methodological Details**:
   - Provide a detailed explanation of the DOE, including the rationale for selecting specific concentrations of quicklime and waterproofing agents. Justify the choice of 27 groups and the criteria for material selection.
   - Clarify the efficacy coefficient method by providing the mathematical formulation, assumptions, and how weighting coefficients were determined.
   - Include a section on quality control measures during sample preparation and testing to ensure reproducibility.
3. **Improve Data Presentation**:
   - Ensure all tables and figures are complete, with clear captions, axis labels, and statistical comparisons (e.g., ANOVA or t-tests) to validate significant differences between groups.
   - Revise incomplete or poorly formatted tables (e.g., Table 5) and figures (e.g., particle gradation diagram). Provide a summary table comparing the performance of all 27 groups across the tested indicators.
   - Include error bars or confidence intervals in graphical representations to indicate data variability.
4. **Address Environmental Factors**:
   - Expand the discussion to include the coupled effects of temperature fluctuations, humidity, and groundwater dynamics, as suggested already. Consider conducting additional experiments or referencing relevant studies to address these factors.
   - Provide a more detailed explanation of how the freeze-thaw cycle test simulates real-world conditions in Kaifeng.
5. **Correct Formatting and Language**:
   - Thoroughly proofread the manuscript to correct grammatical errors, awkward phrasing, and OCR-related issues (e.g., "5.5.5.5" or "$\mathrm{J} \mathrm{J} \mathrm{J}$").
   - Ensure consistent formatting of chemical names (e.g., "styrene-acrylic emulsion" instead of "styrene-acylle") and equations. Verify that all mathematical expressions are correctly rendered.
   - Complete and standardize the reference list, ensuring all citations include necessary details (e.g., DOIs where available).

**Specific Comments**

- **Abstract**: If a graphical abstract has been added , ensure it is clear and visually appealing. The written abstract should concisely summarize the study's objectives, methods, key findings (e.g., 3.9% water absorption for the 5% sodium methylsilicate + 7.5% quicklime group), and implications.
- **Introduction**: Strengthen the literature review by citing more recent studies to contextualize the research gap.
- **Methodology :** Provide a flowchart or diagram to illustrate the experimental workflow, including sample preparation, testing procedures, and data analysis.
- **Results :** The claim of a 3.9% water absorption rate is significant but needs statistical validation. Discuss why sodium methylsilicate outperforms other waterproofing agents in greater detail.
- **Discussion :** Elaborate on the practical challenges of applying these repair materials in situ, such as cost, scalability, and compatibility with existing structures.
- **References**: Ensure all references (e.g., [1], [25], [40]) are complete and follow the journal's formatting guidelines.

**Conclusion**

The manuscript addresses an important topic in the conservation of earthen heritage sites and provides valuable insights into the performance of high-fluidity repair materials. However, significant revisions are needed to improve clarity, methodological rigor, data presentation, and language quality. Addressing the above recommendations will enhance the manuscript's scientific impact and suitability for publication in *PLOS ONE*.

**Recommendation**: **Major Revisions**
