# Supplementary material for: Study on repair materials and technologies for addressing crack-related damage in the Earthen City Wall of Kaifeng
Source: PLoS One. 2025 Oct 27;20(10):e0334834. doi: 10.1371/journal.pone.0334834 (PMC12558513; doi:10.1371/journal.pone.0334834)
Supplement: Attachment — Submitted filename: Reviewer Comments for Manuscript.pdf [file pone.0334834.s001.pdf]

Research on Repairing Materials and Techniques of Cracks in Kaifeng Earth Wall

Decision: Major revision

Detailed Comments to the Authors

The manuscript presents a valuable study on the repair of cracks in Kaifeng's earth walls using various materials, but there are several areas that require significant improvement before publication. One major concern is the lack of a clear justification for the research's novelty. While the introduction provides a detailed background on the deterioration mechanisms of earth walls (Page 2, Lines 10–38), it does not sufficiently differentiate this study from previous works. Many references (e.g., Cui et al. [3,4], Zhang et al. [8,9]) discuss similar approaches using strengthening agents, but the authors fail to explicitly state how their methodology or findings contribute to advancing the field. The manuscript would benefit from a stronger argument on what makes this study unique, such as the combination of repair materials or the specific application of the efficacy coefficient method.

The methodology section lacks crucial details that would allow replication. For example, while the authors describe the preparation of soil samples and repair materials (Page 6, Lines 12–40), they do not provide sufficient information about the curing conditions, environmental parameters, or the exact procedure for mixing and applying the materials. Additionally, while the freeze-thaw cycle test is mentioned (Page 12, Lines 4–18), the manuscript does not specify the temperature range, cycle duration, or the reasoning behind the number of cycles chosen. Given that freeze-thaw durability is a key performance indicator for long-term crack repair, these missing details significantly weaken the reliability of the results. The authors should provide explicit temperature settings and cycle descriptions, referencing standard testing protocols where applicable.

The discussion of results lacks depth in terms of explaining the chemical and physical interactions between repair materials and the soil. For instance, while the manuscript explains that sodium methylsilicate forms a hydrophobic film (Page 10, Lines 30–38), it does not explore whether this film affects the soil's permeability or long-term stability. Additionally, the interaction between quicklime, urease, and urea in forming calcium carbonate (Page 7, Lines 20–38) is described, but the implications of this reaction on the material's porosity and potential shrinkage are not addressed. Furthermore, no information is provided on possible secondary effects, such as efflorescence, which could impact the aesthetic and mechanical properties of the repaired cracks. A more detailed chemical discussion is needed to strengthen the conclusions.

There are several inconsistencies and grammatical errors throughout the manuscript. For instance, "methicosilicate" appears multiple times (Page 3, Line 20; Page 8, Line 5) but is likely meant to be "methylsilicate." Similarly, "waterborne polyurethane" and "water-based polyurethane" are used interchangeably (Page 3, Line 15; Page 9, Line 30), which could confuse readers. Terminology should be consistent throughout the paper. The abstract (Page 1, Lines 10–25) also contains awkward phrasing, such as "the repair material does a great job fixing the simulated crack," which should be revised for a more formal academic tone. Furthermore, certain sentences

are overly informal, such as "this stuff is pretty strong" (Page 2, Line 14), which should be rewritten in a more precise scientific manner.

Figures and tables require improvements in clarity. Some figures, such as the particle gradation diagram (Page 7, Figure 1), lack clear axis labels, making it difficult to interpret trends. Tables summarizing experimental data should include statistical comparisons to highlight significant differences between groups. Additionally, some references in the bibliography appear incomplete or improperly formatted. For example, certain citations (e.g., Page 19, References [12,13]) lack full journal details and DOI links. Ensuring correct formatting and consistency across citations is essential for maintaining academic credibility.
